# The Central Committee's Bill

**Published:** 1919-07-05

**Authors:** 


### NURSE REGISTRATION.

### THE CENTRAL COMMITTEE'S BILL.

### Debate in the House of Commons,

### FRIDAY, JUNE 27.

THIS Bill as amended (in the Standing Committee) was considered.

CLAUSE 2.—(INTERPRETATION.)

"The Council" means the General Nursing Council for the Training and Registration of Nurses in the United Kingdom;

The term "registered nurse" means a woman nurse who is for the time being registered in the woman nurses' general register; the term "registered male nurse" means a male nurse who is for the time being registered in the male nurses' supplementary register; the term "registered mental nurse" means a mental nurse who is for the time being registered in the mental nurses' supplementary register;

The term "Local Government Board" means in relation to institutions in Scotland and Ireland, the Local Government Board for Scotland, and the Local Government Board for Ireland respectively.

**Colonel Burn:** I beg to move, after the word "register" ["supplementary register"] to insert the words,

"the term 'registered children's nurse,' means a children's nurse who is for the time being registered in the children's nurses' supplementary register."

**Major Barnett:** I beg to second the amendment.

Amendment agreed to.

CLAUSE 4.—(CONSTITUTION AND APPOINTMENT OF COUNCIL.)

(1) The Council shall consist of forty-two persons to be appointed or elected as follows:—

(*a*) Three persons to be appointed by the Privy Council;

(*b*) Four registered medical practitioners, two to be appointed by the Local Government Board for England, one for England, one for Wales, one by the Local Government Board for Scotland, and one by the Local Government Board for Ireland;

(*c*) Three registered medical practitioners to be appointed by the British Medical Association, one to be resident in England, one to be resident in Scotland, and one to be resident in Ireland;

(*d*) One registered medical practitioner to be appointed by the Medical-Psychological Association;

(*e*) One registered medical practitioner to be appointed by the medical superintendents of fever hospitals approved by the Council as training schools for nurses in fever nursing;

(*f*) Four persons to be appointed by the nurse training schools attached to hospitals approved by the Council; two by the nurse training schools in England and Wales, one by the nurse training schools in Scotland, and one by the nurse training schools in Ireland;

(*g*) Eighteen registered women nurses to be elected as the direct representatives of the women nurses in the General Register; eight to be elected by the nurses registered in England, two by the nurses registered in Wales, four by the nurses registered in Scotland, and four by the nurses registered in Ireland: Provided that of the eight elected by the nurses registered in England two at least shall be past or present matrons of nurse training schools attached to hospitals approved by the Council, one of whom shall be registered in the General Register as "also trained in fever nursing"; of the four elected by the nurses registered in Scotland one at least shall be a past or present matron of a nurse training school attached to a hospital approved by the Council; and similarly of the four elected by the nurses registered in Ireland, one at least shall be a past or present matron of a nurse training school attached to a hospital approved by the Council;

(*h*) Two registered male nurses to be elected as direct representatives by the male nurses' register;

(*i*) Two registered mental nurses to be elected as direct representatives by the nurses registered in the mental nurses' register;

(*j*) Two registered women nurses to be appointed by the Royal British Nursing Association;

(*k*) Two registered women nurses to be appointed by the College of Nursing, Limited:

Provided that on the first constitution of the Council there shall be thirty-one persons appointed as follows:—

(*a*) Four persons to be appointed by the Privy Council, not being registered medical practitioners, or nurses, or persons engaged in, or associated with, the regular direction or provision of the services of nurses;

(*b*) Four registered medical practitioners, two to be appointed by the Local Government Board for England, one for England and one for Wales, one by the Local Government Board for Scotland, and one by the Local Government Board for Ireland;

(*c*) Four registered medical practitioners to be appointed by the British Medical Association, one to be resident in England, one to be resident in Scotland, one to be resident in Ireland, and one to be resident in Wales;

(*d*) One registered medical practitioner to be appointed by the Medico-Psychological Association;

(*e*) Eighteen women nurses, of whom eight shall be resident in England, two in Wales, four in Scotland, and four in Ireland, to be nominated, subject to the approval of the Local Government Board for England and Wales, the Local Government Board for Scotland, and the Local Government Board for Ireland, respectively, by the following bodies, and in the following numbers:—

One by the Queen Victoria's Jubilee Institute for Nurses;
One by the Asylum Workers' Association;
One by the North Wales and South Wales Nursing Associations;
Four by the Royal British Nurses' Association;
Four by the College of Nursing, Limited; and
Seven, representing the following societies of trained nurses, by the Central Committee for the State Registration of Nurses:—

(1) The Matron's Council of Great Britain and Ireland;
(2) The Society for the State Registration of Trained Nurses;
(3) The National Union of Trained Nurses;
(4) The Fever Nurses' Association;
(5) The Scottish Nurses' Association;
(6) The Irish Nurses' Association;
(7) The Irish Nursing Board.

and the persons so appointed shall hold office for a period of two years and during such period shall form a Register of persons entitled to be registered under this Act.

(2) The members of the Council shall appoint one of the members of the Council to be President of the Council, and also one of the members of the Council to be Vice-President of the Council.

(3) The Lord President of the Council shall, as soon as may be after the commencement of this Act, take such steps as he deems advisable for constituting the first Council and securing the appointment of members thereof, and for summoning the first meeting of the Council.

**Colonel Burn:** I beg to move, in Sub-section (1), to leave out the word "forty-two," and to insert instead thereof the word "forty-five."

**Major Barnett:** I beg to second the amendment.

Amendment agreed to.

**Mr. W. Graham:** I beg to move to leave out the word "forty-two," and to insert instead thereof the word "forty-eight."

The object of this amendment is to make provision for the inclusion of representatives of the local authorities. That can only be done if the number is increased to forty-eight.

**Mr. Deputy-Speaker** (SIR E. CORNWALL): The House has already decided to insert the word "forty-five."

RESENTMENT AGAINST FENWICK BILL.

**Lieut.-Commander Astbury:** I rise to move, in Subsection (1), to leave out paragraphs (*a*) to (*k*), inclusive.

This amendment is in no sense a wrecking amendment. Every member in this House is in favour of the registration of nurses. We know what a noble army these women have been. We know how badly they have been paid in the past. We know their long hours of work, longer than those of any other profession in the country. And anything that can be done for their benefit or can raise their status will have the support of this House. But in trying to get them that support we must see that the Council which will govern his body is an equitable Council, fair to the nurses and fair to the public. I am sorry to have noticed during the last few months, from the different circulars which members of this House have been receiving, that there is a very strong feeling of resentment between different bodies of nurses with regard to this Bill. If it were possible for the Central Committee to withdraw this Bill and for the Government themselves to bring in a Bill, that would be the best course to pursue. Even if this Bill goes through this afternoon, it will not give the

**Nurse Registration—(*continued*).**
satisfaction which we want to give to the whole nursing profession in this country; and I would ask your ruling as to whether I am in order in asking the promoters of this Bill to withdraw it?

**Mr. Deputy-Speaker:** The hon. member proposes to move an amendment, and he cannot raise this point of order on his own amendment.

The Minister of Health Demonstrates.

**Dr. Addison:** Perhaps it may save time if, by leave of the House, I make a statement now. The Government have considered this matter carefully since the Bill passed through Committee. They arranged for meetings with the various parties who are interested in this Bill and another Bill of quite a different character dealing with the same subject which has been introduced into the House of Lords, and we have had conferences with them with the view of discovering whether a sufficient common measure of agreement could be reached by which we could obtain a Bill which would give effect to the registration of nurses, which, I think, by common consent, is regarded as most necessary and desirable. I am sorry to say that the results of these conferences have convinced me against my will that such agreement is not attainable. I think it arises from the fact that, whilst everybody agrees that the registration of nurses is desirable and necessary, it was quite clear in the conferences that those who are interested in the two Bills were not by any means agreed, nor were they likely to agree, as to what was implied by registration. It was quite clear that in some quarters it was thought that the authority responsible for registration should deal with the conditions of training—not only the standard arrived at, but the conditions and conduct of training. If you take the parallel case of the General Medical Council, you will find that, while it lays down the standard attainable, it does not conduct the medical schools. The conduct of the medical school is an educational function. The examining bodies are charged with the responsibility of seeing that their candidates receive a certain minimum standard of training and that they reach certain medical attainments. But the educational practices of these bodies are not, of course, controlled in any way by the Council. As educational bodies they receive grants from the Board of Education, and the same would apply with respect to nurses. Another difficulty which has been made clear by the conferences is that it appears to have been thought by some that as an essential part of the registration the body responsible for the Register should control the conditions of employment, which, again, is a function quite apart from the semi-judicial purpose of such a body. The conditions of employment of nurses or midwives or anybody else are matters of which, no doubt, we shall have to take cognisance when the time comes—I hope it will be shortly—when we have to make grants. It may be that the conditions of employment will then have to be considered. I quite agree with those who attach importance to the conditions of the employment of nurses. Nurses have been scandalously underpaid and grossly overworked, and it is about time that proper or improved conditions of employment were secured for them. Still, that is not the function to be exercised by a semi-judicial body which has to decide who is to be on a Register. I found that the differences on these and kindred subjects were so great that it was quite out of the question to bring about agreement. The controversy appears to have been unfortunately mixed up with personal and sectional issues which cannot be reconciled.

Proposed Government Action.

I have consulted the Government, and I am authorised to say, both in respect of this Bill and of the other Bill referred to, that neither of them is a proper Bill, nor do I see any chance of making either of them a proper Bill, to carry out what we think should be the terms and purpose of a measure dealing with nurses' registration. With the best will in the world, it does not seem to be practical to make this a Bill which will secure passage into law. Therefore I would suggest, whether in the interests of expediting nurses' registration those concerned in this Bill and in the other might think it wise to drop them, and I will undertake at the earliest possible time, on behalf of the Government, to introduce a measure providing for the registration of nurses. As far as possible, I will consult them on the terms of the Bill as to its general and main purposes. Whilst on the one hand we cannot saddle a registration authority with duties that do not properly belong to it, nor can we promote the interests of any sectional institution, on the other hand we will deal with public interests, we will take those concerned into our confidence, and as far as possible I hope they will concur in what we propose. We are prepared to make ourselves responsible for such proposals to Parliament, and to give effect to registration as soon as we can, subject to the exigencies of the Session. You may take that as a *bonâ-fide* pledge. On that understanding, I think it might be for the convenience of the House if both these Bills did not go any further. I should like to say that we are very cognisant of the tact and patience shown by the hon. and gallant member who is in charge of this Bill, and of the interest he has shown in nurses' registration. I fully recognise that. But from what I can learn, if this Bill does go to the Report stage to-day the chances of it finding its way to the Statute Book are very small indeed. The method I suggest is, after all, the shortest way and the best way to the end in view.

Government of the Nurses by the Nurses.

**Lieut.-Commander Astbury:** I beg to move, in Subsection (1), to leave out paragraphs (*a*) to (*k*) inclusive, and to insert instead thereof the words, "namely, one person to be appointed by the Privy Council, one person by the British Medical Association, two persons by the College of Nursing, Limited, one person by the Royal British Nurses' Association, and thirty-seven nurses by election on the part of all nurses duly registered, the first election to take place on the expiration of two years from the passing hereof."

I must say I have listened to the speech of the right hon. gentleman with some disappointment. I thought there might be a chance of carrying out my suggestion. With regard to Clause 4 of the Bill I am going to submit that the Council for governing the nurses should be a Council composed mainly of nurses. A democratic body, on the principle of "government of the nurses by the nurses." We know that the supporters of the Bill before the House have made several charges against the Bill which is at present in the other House, or rather charges against the College of Nursing. One grave charge has been made that the reason why the College of Nursing have got such a large number of nurses on their register, namely, over 14,500, and funds to the extent of £40,000, has been that they have been blackmailing nurses to sign the register and held out the promise that they should be placed on the first State Register. It is needless for me to say that that is quite untrue, because the College could not have promised that. It is also said that the College of Nursing was not in favour of State Registration and that it is only the Central Committee that is in favour of State Registration. I should like the House to know that at the second meeting held by the College of Nursing in London State Registration of nurses was put forward as a definite part of their programme. At the first meeting there was not a unanimous agreement among the nurses on the subject, and the College of Nursing, being mainly an educational body, did not at that moment adopt the system of State Registration. But, as I have pointed out, at their second meeting held in London they finally adopted State Registration of nurses as a definite plank in their platform. Moreover, a great many members of the then Central Committee went over

**Nurse Registration**—(*continued*).

to the College of Nursing Association. It would seem to me to be nothing more or less than an injustice that of the eighteen places allotted to women nurses fourteen should be offered to societies, eleven of which are allied to each other and who represent only a small section, less than 4,000 members, of the nursing profession, whilst four only are allotted to the College of Nursing. The British Nurses' Association claims to have a membership of 30,000, but we have never been able to see their Register. Registers are in existence, but those Registers are not open to the public, whereas the Registers of the College of Nursing, with its membership of over 14,500, are open to anyone who cares to see them. We have no idea whatever whether the British Nurses' Association has a membership of 30,000. Even if they have, we do not know whether those are certified trained nurses, and, in fact, we know nothing whatever about them. Again, 8,000 belong to nine other societies mentioned in the Bill, and they vote several times in the different societies. I should like to point out also that the number of matrons belonging to one of those societies is not known. On the other hand, we do know that over four hundred matrons do not belong to that society, and if we take the number of matrons in this country altogether there are not many more than four hundred, so that the number belonging to this society is infinitesimal. We consider that the Council should be composed mainly of nurses, and that it should be a democratic body and not an autocratic body as it is at present. Surely it should be the nurses who should govern themselves.

An Undemocratic and Perverse Council.

**Major Hurst:** I beg to second the amendment.

The object of it is to substitute for the very undemocratic character of Clause 4 a permanent Council for the Registration of Nurses which shall consist mainly of nurses. By the amendment, thirty-seven out of the forty-two members of this Council will be nurses. That represents what any profession must look to as the goal of its members—government of the nurses for the nurses by the nurses. Our complaint against Clause 4 as it is now drawn is that the permanent Council for the Registration of Nurses is undemocratic, is unrepresentative, and is perverse. By Clause 4 only eighteen of the members of this permanent Council are to be nurses out of a total of forty-two, and out of that eighteen only eight are to be representatives of the general body of nurses of this country, and that number is whittled down again to six by the proviso that two of those eight must necessarily be matrons. A further complaint against the Clause is that of the nominated members of the Council, twenty-four in number, only two represent the College of Nursing with its membership of over 14,000, or something like two-thirds of the nursing profession of this country. It does seem an absurd thing that this highly representative College should have only two of the forty-two nominated members. We say Clause 4 is undemocratic, and we want the Council to be undemocratic. We also say that Clause 4 as it stands does not supply adequate representation in accordance with the wishes of the nurses of this country. Mention has been made as to the numbers who are supporting the Bill as it now stands and who support the amendments which we wish to bring forward. I submit that is to a very great extent material in arriving at a decision whether to accept the Bill as it now stands or the amendments. In the Memorandum which accompanies this Bill something like 30,000 medical practitioners and nurses are claimed as supporting the Bill in its present form, but when the House bears in mind that of that 30,000 who are alleged to be supporting this Bill 22,000 are doctors, they will see what a small residue is left of nurses, and the whole of the British Medical Association, consisting of the great body of doctors of this country, are included in that figure of 30,000. So far as I have been able to find out, there has been no express opinion given by the doctors of this country with regard to the superior merit of this Bill over the Bill pending in another place. On the contrary, all the doctors I have come across speak in the very highest terms of the College of Nursing and disapprove of this Bill in its present form.

A Ludicrous Argument.

Not only are the great majority of nurses against this Bill in its present form and in favour of the amendments which we are moving, but also the flower of the nursing profession is distinctly against the Bill. Speaking for my own district—and I have the honour to represent a district in which a large proportion of the nurses of Manchester live and vote—I can say that all the leading representatives of public opinion among the nurses there are in favour of the College of Nursing scheme, and do not approve of this Bill in its present form. I am told, in answer to that, that that is the upper crust of the profession, and ought not to count. But that is a ludicrous argument. If you wished to take the opinion of the Bar you would not exclude the opinion of King's Counsel, and if you are going to take the opinion of the medical profession you would not exclude the opinion of consultants. And I think the House ought to attach very great weight to the representations which have been made in the last few months to the effect that the leaders of opinion among the nurses do not wish to see Clause 4 in its present form become part of the law of the land, greatly as they favour the idea of registration. A very large proportion of medical men and laymen who are associated with the government and management of hospitals are hostile to this Bill in its present form, and particularly to this Clause. I have been told we ought to disregard that opinion because it is not the opinion of nurses, but where you have, as in the present case, the opinion of the nurses mainly in favour of the College of Nursing Bill, and also the same opinion expressed by those eminent men in the medical profession who are most keenly interested in the education and organisation of nurses, surely great weight must attach to their opinion; and there, again, the doctors I have come across who are most closely associated with the work of education and organisation in the medical and nursing professions are all of one opinion. Clause 4 in its present form is not calculated to attract nurses to be registered at all. There is no magic in registration. If this Bill or any other Bill with regard to the registration of nurses passes into law no penalties are going to be placed on nurses who do not register themselves. They will still be able, if otherwise qualified, to act as nurses and to wear nurses' uniform. The whole point of legal sanction to registration is to attract nurses not merely to become qualified but also registered.

Registration Must be Made Attractive.

There is nothing in this Bill holding out any attractions to nurses to become registered nurses such as we all want to exist. We want to see this attraction, but the Bill holds out no such attraction. In the scheme which is now pending in another place, and which has the support of the College of Nursing, there is something much more than mere registration held out to attract nurses on to the Register. There is the Nation's Fund for Nurses available. There is the utilisation of the existing Register of the College of Nursing; and, in fact, there is a large and hopeful scheme for putting the nursing profession on a much more highly organised and much more educational basis than it is at the present time. It was disappointing to hear from the Minister of Health that he cannot accept the College of Nursing scheme in its present form, and also that he regards registration as something absolutely apart from the question of organisation and education. We who are supporting the amendment do not look upon registration in that light at all. We wish to see it something much more than writing a name down in a book and paying a small subscription. We want to see it made a step-

**Nurse Registration**—(*continued*).

ping-stone to a higher organisation and more continuous and more permanent system of education than at present exists in the nursing profession. For these reasons I am supporting the amendment, although I do not regard it as a perfect amendment, but I have this hope in supporting it, that, by showing by our votes the way in which we regard Clause 4 as it stands, we may do something to induce the hon. member for St. Pancras (Major Barnett) to withdraw his Bill in favour of a Government Bill, which is, after all, what all true lovers of the cause of Registration for Nurses must wish to see. We are not trying to wreck this Bill. Nothing is further from our minds than to defeat Registration, which undoubtedly must be desired by all right-thinking men. Nurses have as much right to be registered as have barristers, solicitors, doctors, or any other occupation. We are not out to wreck the aims of this Bill, but, in asking for the substitution of new Councils for the Councils which are proposed in this Bill, we are trying to urge upon the House and through the House upon the Government the need to bring in the right sort of Bill which will satisfy all these different sects among the nurses, so that the organisation, the education, and the registration of this great and deserving profession may be placed on sound and permanent lines.

Fenwick Party Refuse to Amend.

**Major Barnett:** I am sorry I cannot accept the amendment. I am quite prepared to accept the assurance of the mover of the amendment that it is not intended to be a wrecking amendment, but I can assure him that the effect of it would be to wreck the Bill at this stage of progress, when private members have only got two Fridays after Whitsuntide, and this is one of them, and we have only got up till five o'clock to deal with these amendments. If I were to accept this amendment, which goes to the whole root of the Bill, I say it would be fatal to the measure as it stands, and as it has gone through Committee, and therefore I must oppose it. The hon. gentleman who moved the amendment appealed to me to drop the Bill, and then to the Government to pledge themselves to introduce a measure. A certain pledge has been given by my right hon. friend the Minister of Health, but I am bound to say that I am not prepared to commit *hari kari* even at the suggestion of my right hon. friend. This is a Private Members' Bill. I have never asked for the assistance of the Government. We have made every attempt to meet the views of the College of Nursing. On the question of representation, they had originally only two representatives on the Council, as against twelve, but that has been increased in Committee to four as against eleven, and I have gone further to-day in order to make it eight as against eleven, and it is our Bill. That offer, I think, I am not committing a breach of confidence in saying, is considered by the Government as a fair offer, and it has been refused. I stand by the Bill as it is. I am not going to discuss this amendment on its merits, and I ask my hon. friends not to discuss it on its merits, but, if it goes to a division, to vote it down.

**Lieut.-Colonel Mesey-Thompson:** I rise to support the hon. member who has just sat down in his desire to carry this Bill through. With regard to the amendment, it is said that it is brought forward on democratic grounds, but nothing could be more undemocratic than the suggestion of the hon. and gallant member (Major Hurst) with regard to the wearing of uniform. I certainly shall support the hon. member opposite. I quite agree that if you were to bring in amendments of this kind they might be multiplied to a great extent.

Manchester Opinion in Favour of College.

**Major Nall:** I rise to support the amendment. I am extremely sorry that the hon. member turned down the offer of the Minister of Health. I hold no particular brief for any body, but I support this amendment because I feel that the Bill as it stands is absolutely undemocratic. I am extremely sorry the authors of the Bill have refused to drop this Bill, and bring in a measure based on careful consideration of all the different claims. I must press all the amendments that stand in my name, in order that we may to some extent modify the Bill, and remedy some of the defects which some of us feel strongly exist at present. I entirely associate myself with the remarks of my hon. and gallant friend about the feeling in Manchester and the Lancashire district. That feeling is very intense. Of all the letters I have had from that district, I think only one is in favour of the Bill as it stands; all the others oppose it. Only a few months ago in one of the military hospitals I talked to a good many of the nurses there about this question, and I found very little favour with the position taken up by the Central Committee. During several months in the Manchester Hospital I must say that practically the whole opinion expressed to me by nurses in that institution was in favour of the College. Although I hold no brief for the College, I do feel that we ought to pay some attention to feelings so strongly expressed, and we ought to alter this Bill to meet what, at any rate in the Lancashire district, is the overwhelming body of opinion amongst the nursing profession.

Where do Nurses Come In?

**Mr. Hailwood:** I wish to support the amendment, and I do so, not that I wish to oppose the principle of the Bill, but rather to make the Bill conform to the title. According to the title of the Bill one was given to understand that it was a Nurses' Registration Bill. Under Clause 4, three persons are to be appointed by the Privy Council, four are to be registered medical practitioners, two to be appointed by the Local Government Board for England, one for Ireland, and so on; three registered medical practitioners are to be appointed by the British Medical Association, one registered practitioner is to be appointed by the Medico-Psychological Association, and then one by the medical superintendents of fever hospitals; and four persons are to be appointed by the nursing-training schools. In this way we have persons appointed from different associations who are not nurses at all. We do want State Registration of nurses, but if we are to have State Registration of nurses it should be for nurses by nurses. That is the reason why our amendment increases the number of nurses to be elected by nurses themselves to thirty-seven, and surely in these democratic days it seems preposterous that the outside medical associations should be brought in to control the way in which nurses should be registered. I do not know whether the medical profession would be prepared to accept nurses on their Council to say what shall constitute a medical man, and what qualifications he should possess before he should be registered. I do hold that it would be perfectly ridiculous to expect nurses to control the medical profession, and I strongly object to the medical men being brought into this Bill in order to control the nurses. I am quite confident that the nurses understand their profession better than the doctors do, and I contend that the nurses should be given full power in order to frame their Council, their registration, and their regulations as they think fit. That is the reason why I support the amendment.

An Appeal to the Government.

**Mr. G. Thorne:** I rise only to appeal to the Government. We have all heard the statement made by the Minister of Health, who has indicated that he proposes to bring in a Bill. The promoter of the Bill, exercising his undoubted right, has refused to withdraw this Bill. Consequently the matter is going on for discussion, and a motion is now before the House by way of amendment. Surely the House is entitled to some advice from the Government as between the two sides in regard to this amendment. I should like, under these circumstances, to hear what the Government have to say in regard to the amendment before us.

**Nurse Registration**—(*continued*).

**Mr. Sugden:** In supporting the amendment I am particularly desirous of associating myself with the thought of compromise. I feel that whilst one section of the great nursing fraternity may have had, at some time, some power, and may have dominated the policy of the Council of that very great profession, I do say that in the second suggestion we have put before us something may be done to equalise what has been an overbearing consideration. Therefore I support the amendment of the hon. and gallant gentleman, and I suggest if the mover of the Bill will further consider that addition, it may be possible to arrive at some agreement. I feel that after what the Government have proffered, to undertake to introduce a Bill, it will not be possible in this present Session to carry this forward, and this great profession desires immediate consideration and action. If this Bill is further amended, it may be possible for the hon. and gallant gentleman, and those with whom he is associated, to accept the further suggestion.

A Futile Discussion.

**Major Earl Winterton:** I think it is desirable to have a reply on the point put by the hon. member for Wolverhampton. I do not know whether the position was brought out very clearly in the speech of the Minister for Health, but there have been negotiations going on with regard to this measure. This amendment has been pressed upon the Minister, but it has been impossible to arrive at an agreement while the two present Bills—this one before us and one in another place—are in existence. For this reason the right hon. gentleman proposes to introduce a Bill of his own, which I can assure the hon. member for Wolverhampton has been pressed upon him by a very large concensus of opinion which is not biased in favour of one proposal or the other. It is hardly fair to the right hon. gentleman the Minister of Health to expect him—for he is very busy at this time—to come down here and reply on a discussion which is bound to be a futile one, because this Bill can never be passed.

The Effect of an Amendment.

**Mr. Lyle:** I want to say only a few words on this particular amendment, because it is not an official amendment by the College of Nursing. I am here to-day representing the College, and I must say I am very sorry to hear that there is no chance of my hon. and gallant friend the member for St. Pancras adopting the suggestion which has been put out by the President of the Local Government Board—that both the Bills referred to should be withdrawn, and the Government should inquire into the whole question and get evidence from all sides, as is really the fair and right thing to do. I can speak for the College of Nursing on this matter. We would have welcomed that course being adopted. We quite realise the two points of view, and the reasonableness of the suggestion put forward by the Health Minister. This was that the Government, no doubt after impartial inquiry, should bring in a Bill of their own. Since that course has not been possible, and the hon. gentleman the member for St. Pancras decided to go forward, after looking at the amendment which has been moved by hon. members from the Manchester district, I think that anybody glancing at the Clause fairly will see that the constitution of the Council, as suggested in the amendment, is entirely preferable to the constitution of the Council as drafted in the Bill. Turn to Clause 4. You find that as at present drafted there are three persons to be appointed by the Privy Council. Under the amendment there is only one person to be so appointed—perhaps enough under the circumstances. However that may be, there is under paragraph (*b*)

"Four registered medical practitioners, two to be appointed by the Local Government Board for England, one for England, one for Wales, one by the Local Government Board for Scotland, and one by the Local Government Board for Ireland;"

As against these four registered medical practitioners, in the amendment we propose one person by the British Medical Association. I submit that any Council, which is going to look after the interests of the nurses and govern the nursing profession, that one person, one medical man, is quite sufficient. I do not really think the nurses want to be or should be burdened with four registered medical practitioners. If you read on, you will see in the amendment there are two persons by the College of Nursing, one by the Royal British Nurses' Association, and thirty-seven nurses by election on the part of all nurses duly registered, etc. You have got thirty-seven nurses by direct election, two more by the College of Nursing, and one by the Royal British Nurses' Association. That is forty nurses in all. I am rather surprised at people who have been telling us day in and day out that their policy is one for nurses, and nobody else, trying to make out that they are the democratic people, and nobody else—that those are the people who object to the Council election on the democratic principle suggested in the amendment.

Thirty-seven or Eighteen.

It seems to me that nothing better for the nurses themselves could possibly be conceived than a Council wherein thirty-seven of them are elected directly instead of as in the Bill, where only eighteen nurses are elected direct. That is one point to which I should like to direct the attention of the House.

There is another point which I also would like to dwell upon. That is, in Clause 3, Sub-section (1), paragraph (*a*), you will note that these very democratic bodies have provided:

"that of the eight elected by the nurses registered in England two at least shall be past or present matrons of nurse-training schools attached to hospitals approved by the Council, one of whom shall be registered in the General Register as 'also trained in fever training.'"

It is provided that in the case of Ireland one at least shall be a matron. So that you have this society insisting that a considerable proportion of those elected by the nurses are to be matrons. That strikes me as very odd, because during the last three or four weeks I have had a good deal of correspondence to meet, and the basis of it all, and of many of the articles which have appeared in a paper which is not very widely read but which deals with nursing from a certain point of view, it is consistently advocated that nurses are cast aside in this matter. The whole object of the articles in the newspapers run by the Central Committee is to make out that the matron is a vixen or a tyrant who drives nurses, and who is everything that is bad; in fact, that the matron is a person whom you might almost frighten your children with by mentioning her name.

Freedom of Choice for Nurses.

It has been said that we who advocate the College of Nursing are trying still more to get the nurses under the thumb of the matrons, but we take an entirely different view. We have been accused of actually disfranchising nurses because we put in the word "person" instead of "nurse." We suggested that nurses should be allowed to elect persons and not necessarily nurses, and for that reason we were told that we are disfranchising the nurse. That statement was made with the object of throwing dust into the eyes of people who do not attempt, and would not be bothered about studying this Bill. It has been a campaign throughout of insinuation and slander, and personally I am very sorry that the course has not been adopted of accepting the compromise suggested by the President of the Local Government Board. Our view is not at all that matrons are undesirable people.

Some of us who have worked in hospitals have the greatest admiration for the matrons, and we think they represent some of the finest type engaged in the noblest profession in the world. I do not wish to bar the matrons.

**Nurse Registration**—(*continued*).

On the contrary, we think the nurses appreciate them and look to them in time of trouble, and a matron has often been more than a mother to many a girl who has come to the hospital. We do not share those views about matrons. We think probably matrons would be elected, but I strongly object to providing that a certain proportion of those representing nurses should be definitely matrons. I think that in these days that is a most extraordinary position to take up, and it is more particularly extraordinary when you read the quarter from which it comes. However that may be, I cannot help thinking that anybody who has studied the two Clauses, the one as at present drawn in the Central Committee's Bill and the other, although this is not an official amendment by the College of Nursing, I think anybody who reads this eminently democratic amendment will agree that of the two it is infinitely to be preferred to the Bill as it stands at present. Therefore, I think the House will agree that the amendment is one for which they ought to vote solid.

THE ONLY REASONABLE COURSE.

**Dr. Murray :** I had no intention of speaking upon this amendment, but I rise to express my regret that the hon. and gallant gentleman in charge of the Central Committee's Bill has not accepted the offer of the Government. I should be inclined to vote in favour of the amendment simply on the ground that the hon. and gallant gentleman has not accepted the very reasonable offer made on the part of the Government and on the part of those supporting the College of Nursing. I am not going to rush in to decide between the two parties to this dispute. Sometimes we show our valour by going into the Lobby when the protagonists on both sides are about, and my thoughts on those occasions turn to poetry—

"How happy could I be with either,"
Were t'other dear charmer away."

That is the attitude that I take up with regard to these two Bills. Some hon. members, however, might be inclined to say,

"A plague on both your houses."

I think it is germane to this amendment, because it goes to the essence of the Bill, that the hon. and gallant gentleman should still consider the offer of the Government. There are two or three ways of dealing with this matter. One is for one side or the other to succeed, but that would not bring peace because the other would feel an aggrieved party. We might try peace by negotiation. We might send both parties into a room for a conference, lock the door, and tell them that they must come to an agreement. I do not know that you would find much when you opened the door. The history of the Bill has shown us that the protagonists will not come to an agreement by negotiation. There is only one reasonable way out of the difficulty, and that is for both sides to agree to allow the Government to bring in a Bill dealing with the whole problem. In that way you would not have one aggrieved party as against the other. Possibly in time the differences that have arisen between these two bodies of nurses would gradually fade away. It would certainly be a very great pity that by the victory of one side or the other the nursing profession should be rent in twain. We are merely ploughing the sands, one of the most unremunerative forms of occupation that I know, and I hope that by five o'clock the sponsors of this Bill will agree to the Government taking the matter in hand and trying to settle their differences in the interests of the nursing profession and the health of the country. One hon. member asked, Why should doctors control the nurses? They do not want to in this respect. They only want to control them in connection with their patients. That is all the control they wish to have. But in the matter of the administrative part of the profession I do not think they want to control the nurses. They prefer that that profession should be conducted on democratic lines.

**Mr. Hailwood :** What I said was that the Central Committee are trying to bring in doctors' control.

**Dr. Murray :** I think it will be admitted that the doctors who, after all, direct the operations of the nurses, should have some say as to the qualifications that are necessary for their registration. That is a reasonable demand.

COURTING DISASTER.

**Captain Ormsby-Gore :** I beg to move,

"That the further consideration of the Bill, as amended (in the Standing Committee), be adjourned."

I wish the hon. member in charge of the Bill would agree to that. I profoundly regret the position in which we find ourselves to-day. We are discussing a Bill to-day which everybody knows is bound to die in the course of the next few weeks. I think the remarks of the last speaker really were arguments in support of a motion such as I am making. We do not want to continue what is, after all, a futile discussion. There is a fight going on between the Central Committee on the one side, and the College of Nurses on the other, as to the registration of nurses. But to go on under these circumstances with this Bill is simply to court disaster. I make this motion in order that we may hear further from the hon. member in charge, in general terms, what he proposes to do with regard to the Bill under existing conditions. I am afraid that, through no fault of his, it is going to be wrecked in consequence of the opposition to it from another section. The amendment now before us is not going to be the end of the matter. There are two Bills dealing with this subject. I regret that the hon. member has not taken the offer of the Government and accepted its undertaking to introduce this Session a Bill to effect the registration of nurses.

**Major Molson :** I beg to second the motion.

I hope the mover of this Bill will withdraw the measure, and that the parties concerned will agree to leave their interests in the hands of the Government. On the last occasion this measure was under debate I supported it very strongly because I recognised the desirability of having a general registration of nurses. I am extremely sorry to find there are so many party differences on this question. I was in the Committee Room when the deputation came, and I was much alarmed to notice the existence of these party interests. I do not like seeing one party up against another in this way. I am not favouring one party more than the other. I do not think there is much to choose between them, but as there is so much feeling in the country I heartily advise the mover of this Bill to withdraw it and let the Government bring in an impartial measure which will benefit the nurses throughout the Kingdom.

THE LADY'S FINAL FULMINATION.

**Major Barnett :** I have sat with patience during the last hour listening to criticisms of myself for not having accepted an offer which has been made by the Ministry of Health. I listened very carefully to that offer, and it simply amounted to a pledge that the Government would look into this matter and deal with it at some future time, not this Session. (Hon. Members: "No.") I do not want to throw the slightest doubt upon their good intentions, but we have had in this House several examples of Government intentions that have not been carried out. I remember a most famous preamble to a Bill dealing with another place and an important constitutional problem, which was regarded as an obligation of honour by the Government of the day which should be carried out, but that obligation of honour was never carried out. Although my hon. and gallant friend opposite appeals to me to withdraw the Bill and to the Government to give some pledge that they will take up the question, nothing in the way of a real pledge has been given. This being one of the two last Fridays after Whitsuntide for private members' business, I felt fully entitled to go on in order to see if we can get the report stage of the Bill. If we can get the report stage of the Bill before five o'clock, it would do no harm to the College of Nursing, Limited, or its Man-

**Nurse Registration**—(*continued*).

chester advocates. In another place the College of Nursing, Limited, by guarantee, sits entrenched. (Hon. Members: "No.") I know something of what I am saying. If this Bill passes line for line as I want it, it would go to another place and the interests of the College of Nursing would be very well looked after there. I suggest that as we have a short time allowed by the Rules of the House, and this is one of the Friday afternoons given to private members' business, we should go on and try to get the report stage. If there had been any real desire on the part of the advocates of State Registration, who in this House have given lip service to the principle, they would have ceased to take the first opportunity of talking out a Bill which is the only Bill that has the slightest chance of becoming law. The other Bill brought in by another place, which has passed its Second Reading, would never come here at all. If it did, it would be laughed out here. A private Bill, promoted by a company limited by guarantee, which is to form the basis of the State Registration of nurses.

**Lieut.-Commander Astbury**: May I remind my hon. and gallant friend that it is not a limited company? It is "limited" by guarantee—quite another pair of shoes.

**Major Barnett**: It has not been dropped yet. It is the College of Nursing, Limited. The Bill which my hon. and gallant friend says has passed has only obtained a Second Reading in one House. I agree that it is undesirable to continue a futile discussion. I only wish to make it quite clear that if this Bill, which the great body of nurses passionately desire, is not going through this House, it is because there is an organised opposition in the interest of another body of nurses. I appeal to hon. members who have moved these amendments—which they do not call wrecking amendments, but which fulfil that purpose—not to oppose the motion to report progress.

CENTRAL COMMITTEE REFUSE THE PATH OF WISDOM.

**Colonel Greig**: I am a keen supporter of the Bill, and I came here in order to support it, but having listened carefully to what has been said, and especially in view of the fact that the Minister for Health has given a definite pledge that a Bill shall be introduced—

An Hon. Member: When?

**Colonel Greig**: The hon. member who is supporting the present Bill can very easily translate that pledge into actual fact by inducing his supporters to meet and agree with the supporters of the other Bill and arrange terms and have what would practically then be an agreed public Bill brought in on the responsibility of the Government which would then meet with even more support than any private members' Bill could meet with. I earnestly suggest that the hon. member should withdraw the Bill. Even if the other Bill is entrenched in the House of Lords and never comes down to this House there is not the least doubt that it would have the smallest chance of success if he takes that course. I therefore earnestly, as a supporter of the Bill, and in the interests of all parties, in order to put an end to this intensive struggle, which does no good to the nursing profession, and is certainly not calculated to do good to the public outside, ask the hon. member to adopt the course that has been suggested so that an early Government Bill can be introduced which will be satisfactory to all parties and to this House, which, after all, is the main body to be consulted. It is not the hon. member's Bill nor the Bill of the hon. members below the Gangway which will eventually pass this House. It will be Parliament's Bill.

THE GOVERNMENT'S DISTINCT PLEDGE.

**Mr. G. Thorne**: I intervened a little while ago when the amendment was before the House, asking the Government to exercise their discretion and give their assistance. They did not give it. Now the situation is the adjournment of the debate. The point of the adjournment was that the hon. member should withdraw his Bill, in view of the Government pledge, but he has indicated that he considers the Government has given no pledge. I distinctly understood the Government gave a pledge, and that was the whole point, as I understood it, of the right hon. gentleman urging the hon. member to withdraw the Bill. If the Government has given no pledge the position of the hon. member is certainly a very difficult one. I ask the hon. gentleman representing the Government to say whether I am right or not that the Government has distinctly promised to bring in a Bill, and that that is the justification for withdrawing this Bill?

**Commander Eyres-Monsell** (Treasurer of the Household): I will give the hon. gentleman an answer, though I should have thought it was sufficiently obvious. The Minister of Health gave a pledge; he said the Government was going to take up this question, and it believed that step would meet the wishes of the great majority of the House and also of the people interested in this most important question throughout the country. That being so, the Government did not intend to take any further part in this discussion, either by vote or by speech, and I cannot help feeling it would be better if the motion was accepted and the further consideration of the Bill adjourned.

THE POSITION IN A NUTSHELL.

**Mr. Lyle: I should like to support the motion to report progress. The more we talk about this matter in the House as to the differences there are, the more difficult it will be to come to agreement when the Minister of Health asks us to come together for the Government Bill. I repudiate the suggestion made by the hon. Member (Major Barnett) that we wish to betray any nurses. We are just as keen and anxious for State Registration as the promoters of this Bill. We also have a duty to perform to the nurses who trust us. We are pledged to support them, and the hon. and gallant Member has only himself to blame if he finds himself in this position. We supported the Second Reading and affirmed our principles in favour of State Registration, but we said that our further support would entirely depend as to how he met our reasonable objections. We have not been met, and he has only himself to blame. I am not in the least afraid that our action will be resented by the majority of nurses. I am sure the majority of nurses will be pleased that their case, which is a noble case, should be taken from the bitter arena of recrimination and put into the hands of a neutral body who will see fair play. My hon. friend tells us of the dreadful things that may happen to us when we get outside, but perhaps he will thank us for saving him from the dreadful things that might happen to him if the Third Reading had been obtained to-day.**

Major Entwistle rose.

**Mr. Speaker**: Is there any use in continuing the discussion?

Question put, and agreed to.

Bill, as amended in the Standing Committee, to be further considered upon Friday next.

